# LC478, a Novel Di-Substituted Adamantyl Derivative, Enhances the Oral Bioavailability of Docetaxel in Rats

**DOI:** 10.3390/pharmaceutics11030135

**Published:** 2019-03-20

**Authors:** Seung Yon Han, Qili Lu, Kyeong Lee, Young Hee Choi

**Affiliations:** College of Pharmacy and Integrated Research Institute for Drug Development, Dongguk University_Seoul, 32 Dongguk-lo, Ilsandong-gu, Goyang-si, Gyonggi-do 10326, Korea; hsyglory@gmail.com (S.Y.H.); luqili220@gmail.com (Q.L.)

**Keywords:** docetaxel, bioavailability, absorption, LC478, P-glycoprotein, inhibition

## Abstract

P-glycoprotein (P-gp)-mediated efflux of docetaxel in the gastrointestinal tract mainly impedes its oral chemotherapy. Recently, LC478, a novel di-substituted adamantyl derivative, was identified as a non-cytotoxic P-gp inhibitor in vitro. Here, we assessed whether LC478 enhances the oral bioavailability of docetaxel in vitro and in vivo. LC478 inhibited P-gp mediated efflux of docetaxel in Caco-2 cells. In addition, 100 mg/kg of LC478 increased intestinal absorption of docetaxel, which led to an increase in area under plasma concentration-time curve (*AUC*) and absolute bioavailability of docetaxel in rats. According to U.S. FDA criteria (*I*, an inhibitor concentration in vivo tissue)/(*IC*_50_, inhibitory constant in vitro) >10 determines P-gp inhibition between in vitro and in vivo. The values 15.6–20.5, from (LC478 concentration in intestine, 9.37–12.3 μM)/(*IC*_50_ of LC478 on P-gp inhibition in Caco-2 cell, 0.601 μM) suggested that 100 mg/kg of LC478 sufficiently inhibited P-gp to enhance oral absorption of docetaxel. Moreover, LC478 inhibited P-gp mediated efflux of docetaxel in the ussing chamber studies using rat small intestines. Our study demonstrated that the feasibility of LC478 as an ideal enhancer of docetaxel bioavailability by P-gp inhibition in dose (concentration)-dependent manners.

## 1. Introduction

Docetaxel, a second-generation taxane, has significant and broad anti-tumor activities against breast, ovary, prostate, non-small cell lung, and gastric cancers [1,2]. An intravenous formulation of docetaxel that is currently marketed (Taxotere^®^, Sanofi SA, Paris, France) shows several drawbacks including non-ionic surfactant-induced severe hypersensitivity reaction [3], skin damage, extravasation catheter-related infection, and potential thrombosis by intravenous injection [4,5]. Oral docetaxel chemotherapy is a desired alternative regimen [5,6] in terms of administration convenience, better patient compliance, and prolonged systemic exposure profile with less fluctuation [7,8,9]. Nevertheless, the low oral bioavailability of docetaxel (less than 5% of the oral dose) impedes its clinical application of oral chemotherapy owing to its physiochemical properties (e.g., low water solubility and membrane permeability) and its physiological barriers (e.g., pre-systemic metabolism via cytochrome P450 (CYP)3A and transmembrane efflux via P-glycoprotein (P-gp)) [2,5,10,11,12,13,14,15].

In pharmacokinetic characteristics of docetaxel, docetaxel is metabolized via CYP3A isoforms and the predominant elimination of docetaxel and its metabolites are biliary and intestinal excretion as systemic clearance pathways of docetaxel [16,17]. In addition, ATP-binding cassette (ABC) transporters (e.g., P-gp, multidrug resistance-associated protein 1 (MRP1), MRP2 and breast cancer resistance protein (BCRP) transporters) in enterocytes or hepatocytes and human solute carrier (SLC) transporters (e.g., organic anion transporting polypeptides (OATP)1B1 and 1B3) in hepatocytes can modulate docetaxel disposition [18,19,20,21].

In tackling the hurdles of a low bioavailability of docetaxel, P-gp (MDR1 or ABCB1) has gained attention. P-gp has been identified to regulate absorption, distribution, and/or excretion of a broad range of more than 300 compounds [22,23,24]. P-gp is highly expressed in tumor cells and normal tissues including intestines, liver, kidney, blood brain barrier, testes, and placenta [22,23]. In particular, P-gp-mediated efflux of its substrate drug from the gut wall into the intestinal lumen hampers oral absorption [25]. At this point, inhibiting P-gp can increase systemic exposure and subsequent pharmacological efficacy in target tissues of its substrate drug [25,26]. Numerous efforts to overcome low oral bioavailability of docetaxel with P-gp inhibitors (e.g., verapamil, cyclosporine, ketoconazole, and quinidine for first-generation; R-verapamil, valspodar, and viridodar for second-generation; and elacridar, zosuquidar, and ONT-093 for third-generation) have been undertaken [2,10,11,19,20,27,28]. Especially, in the development of third generation of P-gp inhibitors, their own pharmacological activities, intrinsic toxicities, non-selectivity, and unexpected pharmacokinetic interactions (e.g., reduction of CYP3A-mediated metabolic activities) of P-gp inhibitors have overcome and they showed sufficient efficacy as P-gp inhibitor in vitro and in vivo [19,20,29,30,31,32]. Thus, the maintenance of sufficient concentration to inhibit P-gp function in the intestine in tandem with non-toxic concentration in other tissues is required for P-gp inhibitor candidates to enhance docetaxel bioavailability.

**Figure 1:**
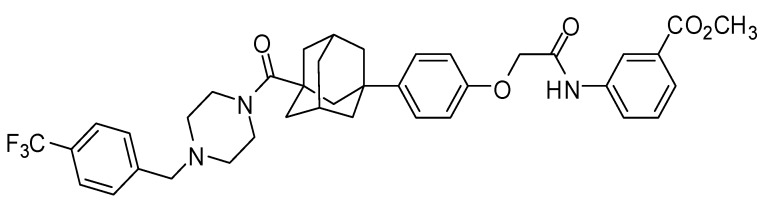
Structure of LC478.

Min et al. [33] reported that LC478 (Figure 1), one of di-substituted adamantyl derivatives synthesized, reversed P-gp mediated efflux activity of palictaxel in P-gp overexpressing MES-SA/DX5 sarcoma cells without intrinsic cytotoxicity. Therefore, we investigated the potential of LC478 for enhancing the oral bioavailability of docetaxel by P-gp inhibition in vitro and in vivo.

## 2. Materials and Methods

### 2.1. Chemicals

LC478 (Figure 1) was prepared following the previously reported procedure [33]. Shin Poong Pharmaceutical Company, Ltd. (Ansan, Korea) supplied the docetaxel trihydrate. Paclitaxel (internal standard for docetaxel analysis using liquid chromatographic tandem mass spectrometry (LC-MS/MS)), dextran (*MW* 65,000), the reduced form of β-nicotinamide adenine dinucleotide phosphate, NADPH as a tetrasodium salt), tri(hydroxymethyl)aminomethane(tris)-buffer, 3-(4,5-dimethylthiazol-2-yl)-2,5-diphenyltetrazolium bromide (MTT), verapamil, and rhodamine-123 were purchased from Sigma-Aldrich Korea (St. Louis, MO, USA). Other chemicals and reagents were high-performance liquid-chromatography (HPLC) grades. 

### 2.2. Cell Culture and Cell Viability Assay

Caco-2 cells were obtained from the Korean Cell Line Bank (Seoul, Korea). The cells were maintained in Dulbecco’s modified Eagle’s medium (DMEM) supplemented with 10% fetal bovine serum, 1% non-essential amino acids, and 100 U/mL penicillin and gentamicin at 37 °C in a humidified 5% CO_2_ atmosphere [34].

The effect of LC478 on cell viability was assessed by an MTT assay. LC478 was dissolved in diluted in 100% dimethyl sulfoxide (DMSO) and diluted with cell culture media. Caco-2 cells were seeded in 96-well plates at 1 × 10^5^ cells/mL. A 100 μL of LC478 in cell culture media was treated on the plates to achieve final concentration of LC478 in the ranges of 0.001 to 100 μM, which was incubated for 24 h. After adding 10 μL/well of MTT (5 mg/L) and incubating them for 24 h, the supernatants of the cultures were removed and replaced with 100 μL of DMSO. The cell viability rate (%) was calculated as the absorbance of treated cells divided by that of control cells. The viability of the control cells was defined as 100%.

### 2.3. Effect of LC478 on P-gp Mediated Efflux of Rhodamine-123, a P-gp Substate, in Caco-2 Cells

To investigate the effect of LC478 on P-gp activity, the transcellular transport activity of rhodamine-123 across the Caco-2 cells was performed with modification of the previous reports [35,36,37,38]. Rhodamine-123 and verapamil were used as a typical P-gp substrate and inhibitor, respectively. Caco-2 cell was seeded at a surface density of 160,000 cells/cm^2^ on polycarbonate microporous membrane inserts in 12-well Transwell plates. They were allowed to grow to confluence for 5 days to obtain higher expressions of P-gp. The transcellular transport activities of doectaxel in Caco-2 monolayers were measured when transepithelial electrical resistance (TEER) values were higher than >200 Ω·cm^2^. Briefly, both apical (A) and the basolateral (B) chambers of each insert were washed twice with 37 °C in Hank’s balanced salt solution (HBSS) buffer with pH 7.4, and were pre-incubated for 30 min. The assay was initiated by replacement of buffer at either the A (0.5 mL) or B side (1.5 mL) containing rhodamine-123 (1 μM) with vehicle, LC478 (1 and 10 μM) or verapamil (10 μM), respectively. At 30, 60, 90, 120, and 150 min, a 200 μL buffer was removed from the receiver compartment and replaced with the same volume of HBSS solution at 37 °C. All samples were stored at −80 °C until the determination of rhodamine-123 using LC-MS/MS analytical method [39].

In addition, effect of LC478 on intracellular accumulations of rhodamine-123 in Caco-2 cells was evaluated by following the modification of the previous reported method [40]. Fifty thousand Caco-2 cells were seeded in 48-well plates and they were allowed to grow to confluence for 5 days to obtain higher expressions of P-gp. When the cells reached to 90% confluency, 200 μL of vehicle, verapamil (0.001–100 μM) or LC478 (0.001–100 μM) was added per well, respectively. After 24 h pre-treatment of verapamil or LC78, cells were washed with phosphate buffer saline (PBS) and 200 μL of 10 μM rhodamine-123 diluted in HBSS with 10 mM HEPES (pH 7.4) was added to each well. After 2 h incubation, the uptake was stopped by aspirating the rhodamine-123/HBSS solution and washing the cells 3 times with ice-cold PBS. Subsequently, cells were lysed with 200 μL of 0.1% Triton X-100 for 30 min at room temperature and 100 μL aliquots were used to measure rhodamine-123 using the LC-MS/MS analytical method [39]. The half-maximal inhibitory constant (*IC*_50_) values of LC478 or verapamil for inhibition of P-gp activity are corresponded to the half-maximal effective constant (EC_50_) for increasing rhodamine-123 accumulation. Data are expressed as a % of rhodamine-123 concentration in control Caco-2 cells (exposed to vehicle), arbitrarily at 100% and are the means of standard error of the mean (SEM) of three independent experiments.

### 2.4. Effect of LC478 on P-gp Mediated Efflux of Docetaxel in Caco-2 Cells

To evaluate the effect of LC478 on P-gp mediated efflux of docetaxel in Caco-2 cells, IC_50_ values of LC478 on inhibition of intracellular accumulations of docetaxel were calculated by the same methods in Section 2.3. Instead of rhodamine-123, docetaxel was adjusted.

### 2.5. Animals

Male Sprague–Dawley rats at 6–9 weeks old (weighing 200–260 g) were purchased from Charles River Company Korea (Orient, Seoul, Korea). The institute of Laboratory Animal Resources of Dongguk University_Seoul (Seoul, Korea) reviewed and approved the experimental protocols involving animals in this study (approval no. IACUC-2015-044, 12 September 2015). All rats were maintained in the same conditions as a reported method [35,37]. 

### 2.6. Pharmacokinetic Studies of Docetaxel with LC478

Early in the morning, rats were anesthetized by intramuscular injection of 125 mg (1.5 mL)/kg of tiletamine HCl and zolazepam HCl mixture. The surgical procedures including cannulating the carotid artery (for blood sampling) and/or the jugular vein (only for intravenous drug administration) using PE50 tubes were conducted under light ether anesthesia and rats were allowed for 4–5 h to recover from the anesthesia before the study began similar to a previously reported method [35].

For the intravenous study, rats were divided into three groups according to LC478 doses (0, 30, and 100 mg/kg LC478, respectively). Two hours before docetaxel administration, oral administration of 0, 30, or 100 mg (5 mL)/kg LC478 (dissolved in the polyethylene glycol 400 (PEG400): Distilled water = 1:1, *v*/*v*) was performed in rats. After 2 h, 20 mg (2 mL)/kg of docetaxel (docetaxel trihydrate dissolved in dimethylacetamide (DMA): Distilled water = 4:6, *v*/*v*) was administered intravenously to the rats. Blood samples (approximately 0.22 mL, each) were withdrawn from the carotid artery at 0 (control), 1 (end of the docetaxel infusion), 5, 15, 30, 60, 90, 120, 180, 240, 300, and 360 min after docetaxel administration. To prevent blood clotting, 0.3 mL of 0.9% NaCl-injectable solution containing heparin (20 U/mL) was flushed into each cannula immediately after each blood sampling. After the blood sample was centrifuged, a 100 μL aliquot of plasma was stored at −70 °C (Revco ULT 1490 D-N-S; Western Mednics, Asheville, NC, USA). The 24 h urine and gastrointestinal tract (its entire contents including feces) samples were prepared and handled following a previously reported method [35]. At 24 h, distilled water (10 mL) was gently flushed into each metabolic cage and the resulting fluid combined with the urine was collected over the previous 24 h. At this time, each rat was sacrificed by withdrawing blood via a heart puncture, and then the entire gastrointestinal tract (including its contents and feces) was extracted and transferred into a beaker. After adding 100 mL of methanol (to facilitate the extraction of docetaxel) into each beaker, the gastrointestinal tract samples in methanol were cut into small pieces and manually stirred. A 100 μL aliquot of the supernatant was collected from each beaker.

For the oral study, after overnight fasting with free access to water, rats were divided into three subgroups according to the doses of LC478 administered, which were the same as in the intravenous study. After 2 h, 20 mg (5 mL)/kg of docetaxel (the same solution used in the intravenous study) was orally administered to the rats using a gastric gavage tube. Blood samples were withdrawn from the carotid artery at 0, 5, 15, 30, 60, 90, 120, 180, 240, 300, and 360 min after oral administration of docetaxel. Other procedures were followed to the intravenous study.

Based on the standard methods [41,42,43,44], we calculated the following pharmacokinetic parameters using non-compartmental analysis (WinNonlin^®^; Professional Edition version 2.1; Pharsight, Mountain View, CA, USA): The total area under the plasma concentration–time curve from time zero to last blood sampling time (*AUC*_last_) or time infinity (*AUC*_inf_) by log-linear trapezoidal extrapolation method [37], terminal half-life, time-averaged total body, renal, and non-renal clearances (*CL*, *CL*_R_, and *CL*_NR_, respectively), mean residence time (*MRT*), apparent volume of distribution at a steady state (*V*_ss_), apparent oral clearance (*CL*/*F*), and apparent volume of distribution during elimination (*V*_z_/*F*). We directly read the peak plasma concentration (*C*_max_) and time to reach *C*_max_ (*T*_max_) from the experimental data. The percentage of the dose excreted into urine up to 24 h (*Ae*_0–24 h_) and the percentage of the dose recovered from the gastrointestinal tract (including its contents and feces) at 24 h (*GI*_24 h_) were calculated from concentrations of docetaxel in 24 h urine and gastrointestinal tract samples versus the administered dose of docetaxel, respectively [37].

The absorbed fraction of oral dose (*F*_abs_) [45], absolute bioavailability (*F*), and relative bioavailability (*F*_rel_) were calculated by the following equations.

(1) $$F\;(\%)=\frac{{\mathrm{AUC}}_{\inf,\;p.o}}{{\mathrm{AUC}}_{\inf,\;i.v}}\times 100$$

(2) $$F_{\mathrm{rel}}\;(\%)=\frac{{\mathrm{AUC}}_{\inf}\;\mathrm{with}\;\mathrm{LC}478}{{\mathrm{AUC}}_{\inf}\;\mathrm{without}\;\mathrm{LC}478}\times 100$$

(3) $$\frac{{\mathrm{GI}}_{24\;h,\;p.o}}{100}=(1-F_{\mathrm{abs}})+(\frac{F}{100}\times \frac{{\mathrm{GI}}_{24\;h,\;i.v.}}{100})$$

The “i.v.” and “p.o.” represented intravenous and oral administration, respectively.

### 2.7. Effects of LC478 on Bi-Directional Transport of Rhodamine-123 or Docetaxel Across Rat Duodenum Using the Ussing Chamber

To evaluate the effect of LC478 on P-gp mediated efflux activity in rat’s small intestine, transport experiments were conducted following the previously reported method [37,46,47]. The incubation medium (pH 7.4) involving 1.2 mM NaCl, 5 mM KCl, 1.2 mM CaCl_2_, 1.2 mM MgCl_2_, 25 mM NaHCO_3_, 1.6 mM Na_2_HPO_4_, 0.4 mM NaH_2_PO_4_, 5 mM d-glucose, and 5 mM D-mannitol was used throughout the transport experiments. After blood sampling was conducted by heart puncture under the light anesthetic condition by a mixture of 125 mg/kg of tiletamine HCl and zolazepam HCl, each control rat was sacrificed by cervical dislocation. The small intestine (2 cm from the proximal part of jejunum) was removed, opened along the mesenteric border and rinsed immediately in ice-cold buffer (pH 7.4) under carbogen O_2_/CO_2_ (95%/5%) bubbling. The specimens were fixed to 0.61 cm^2^ surface area of cells in the Ussing chambers without harming the underlying muscle layer and formed two compartments, mucosal (M) and serosal (S) chambers. One milliliter of incubation medium was spiked into each chamber at 37 °C with continuous oxygenation and carbogen O_2_/CO_2_ (95%/5%) bubbling through each compartment for oxygenation and agitation. Then, 5 µL of verapamil (at a final concentration of 10 µM), LC478 (at a final concentration of 0, 1, or 10 µM) or incubation medium (control) was added to both sides of the chamber. After 30 min of pre-incubation time before the experiments began, rhodamine-123 (at a final concentration of 10 µM) was added to the apical compartment for absorptive (M to S) transport or to the basolateral compartment for secretory (S to M) transport. During the 3 h for the transporter experiment, each 50 µL sample was withdrawn from the acceptor chamber at half-hour intervals and immediately replaced with 50 µL of incubation medium. The concentration of rhodamine-123 in each sample was determined by HPLC analysis [39]. Cumulative corrections were made for previously removed samples and apparent permeability coefficients (*P*_app_, cm/s) were calculated as follows: *P*_app_ = *Q/A*·*c*·*t*, where *Q* is the total amount of the drug permeated throughout the incubation time, *A* is the diffusion area of the Ussing chamber, *c* is the initial drug concentration in the donor compartment, and *t* is the total time of the experiment. Efflux ratios were calculated from *P*_app_ values by: Efflux ratio = secretory *P*_app_/absorptive *P*_app_.

To evaluate the effect of LC478 on the transepithelial transport of docetaxel, the same experiment was performed using docetaxel (1 µM) instead of rhodamine-123.

### 2.8. Effect of LC478 for Disappearance of Docetaxel in Rat Hepatic and Intestinal Microsomes

We investigated the inhibitory mode for the metabolism of docetaxel by LC478 based on a previously reported method [37]. The hepatic and intestinal microsomes were prepared based on a similar reported method [37].

The following constituents were added to a tube: Hepatic (1 mg protein) or intestinal (0.3 mg protein) microsome; 2.5 µL of methanol containing 0.2, 1, 5, 20, 50, and 100 µM docetaxel (a substrate) as final concentrations; 2.5 µL of methanol containing 0, 1, 5, and 10 µM LC478 (an inhibitor) as final concentrations and 50 µL of 1 mM of NADPH dissolved in 0.1 M phosphate buffer of pH 7.4. Adding 0.1 M phosphate buffer (pH 7.4) adjusted the total volume as 0.5 mL, and then the components were incubated at 37 °C using a thermomixer at 500 opm. After incubation of 15 and 30 min for the hepatic and intestinal microsomes, respectively, 1 mL of acetonitrile with 5 µg/mL of paclitaxel as an internal standard added to terminate the reaction. We determined the concentration of docetaxel in each sample by LC-MS/MS analysis [37].

We calculated the kinetic constants (*K*_m_ and *V*_max_) for the docetaxel disappearance using non-linear regression [48] using Sigma plot 10.0 (Systat Software, San Jose, CA, USA). The unweighted kinetic data from rat hepatic and intestinal microsomes were fitted using a single-site Michaelis–Menten Equation

*V* = *V*_max_ × [S]/(*K*_m_ + [S]) (4)

where [S] is the substrate concentration, *V*_max_ is maximal velocity, and *K*_m_ is substrate concentration for half-maximal velocity. The *CL*_int_ was calculated by dividing the *V*_max_ by the *K*_m_.

### 2.9. Effect of LC478 on Rat Plasma Protein Binding of Docetaxel with LC478

Using equilibrium dialysis [37], protein binding values of docetaxel with and without LC478 were measured in fresh plasma from control rats (*n* = 5; each). A 1 mL of the plasma was dialyzed against 1 mL of isotonic Sørensen phosphate buffer (pH 7.4) containing 3% dextran (*w*/*v*) in a dialysis cell (Spectrum Medical Industries, Laguna Hills, CA, USA) using a Spectra/Por 4 membrane (mol. wt. cutoff 12−14 KDa; Spectrum Medical Industries, USA). After 24 h incubation, 50 µL was collected from each compartment and determined using LC-MS/MS analysis of docetaxel [37].

### 2.10. Analytical Methods of Docetaxel and LC478

Concentrations of docetaxel in the samples were determined with a slight modification to the previously reported LC-MS/MS analysis [37]. A 100 µL of a biological sample was deproteinized with 200 µL of acetonitrile containing 25 ng/mL of paclitaxel (internal standard). After vortex-mixing and centrifugation of the above mixture, 10 µL of the supernatant was directly loaded onto a C_18_ column (Symmetry BEH phenyl; 100 mm. ℓ. × 2.1 mm. i.d.; particle size, 3.4 µm; Waters, Milford, MA, USA). The mobile phase consisted of 0.1% formic acid and acetonitrile (50:50, *v*/*v*), which was run at a flow-rate of 0.3 mL/min. The API 4000 triple quadrupole mass spectrometer (ABI/MDS sciex model, Framingham, MA, USA) operated in positive multiple reaction monitoring mode at 50 L/min of nebulizing gas flow, 50 L/min of the auxiliary gas flow, 50 L/min of CAD gas flow and 20 L/min of curtain gas flow. The source temperature was set at 250 °C and the ion spray voltage was 5500 V. The MS/MS transitions of docetaxel and paclitaxel were *m*/*z* [M+H]^+^ 808.5→527.1 and *m*/*z* [M+H]^+^ 854.3→286.2, respectively. The detection limits of docetaxel were 0.1 ng/mL in biological samples with a signal to noise ratio of 3.

Concentration of LC478 was determined using a HPLC-UV system. A 50 µL aliquot of acetonitrile was added to a 50 µL aliquot of biological sample. After vortex-mixing and centrifugation, the supernatant was evaporated (Dry Thermo Bath MG-2200, Eyela, Tokyo, Japan) under a soft stream of nitrogen gas at 50 °C. The residue was reconstituted in 60 µL mobile phase and a 50 µL aliquot of the supernatant was loaded onto a reverse-phase C_18_ column (SunFireTM; 150 mm. ℓ. × 4.6 mm. i.d.; particle size, 5 µm; Waters, Milford, MA, USA). The mobile phase was organic solvent consisting acetonitrile: Methanol at a ratio of 25:45 (*v*/*v*) with 0.3% formic acid, and the flow rate was 1.7 mL/min. The column eluent was monitored at 256 nm. The retention time of LC478 was approximately 7.8 min, and the quantitation limit was 0.1 µg/mL.

### 2.11. Statistical Analysis

When it was necessary to compare the means among the three means for the unpaired data, a Duncan’s multiple range test in conjunction with posteriori analysis of variance (ANOVA) in Social Package of Statistical Sciences (SPSS) was typically used. Statistical significance was considered as a *p* value of <0.05.

## 3. Results

### 3.1. Effect of LC478 on Cace-2 Cell Viability

The effect of LC478 on Caco-2 cell viability was evaluated using an MTT assay. Caco-2 cell was cultured with different concentrations of LC478 from 0.001 to 100 µM for 24 h and the cell viability was 97–100%. Results showed that LC478 was not toxic to Caco-2 cells even at 100 µM for 24 h incubation.

### 3.2. Effect of LC478 on P-gp Mediated Efflux of Rhodamine-123, a P-gp Substate, in Caco-2 Cells

**Figure 2:**
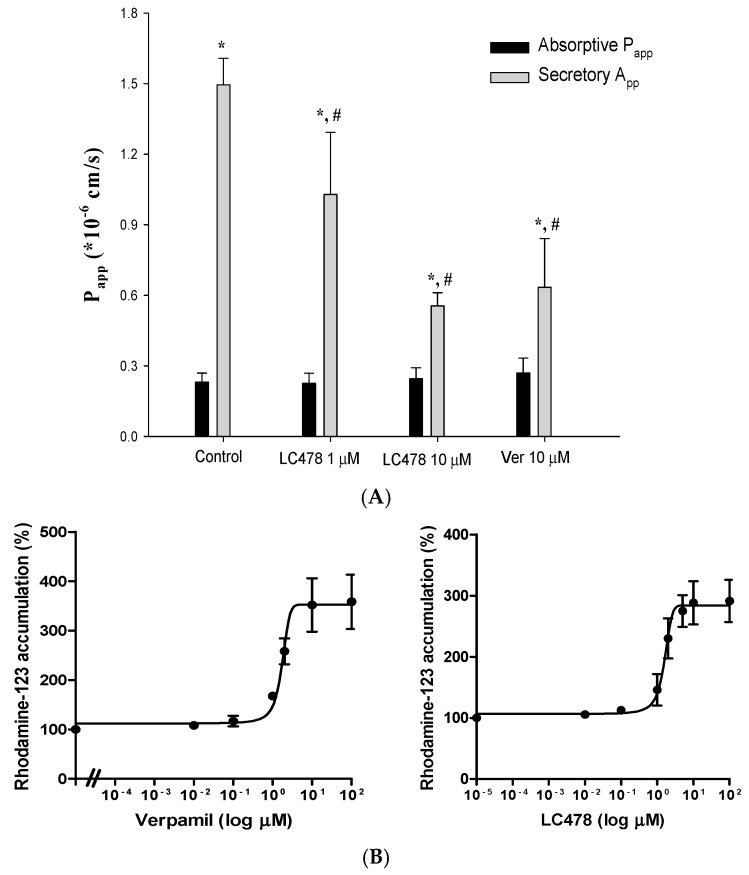
Effect of LC478 on P-gp mediated efflux of rhodamine-123. (**A**) Absorptive *P*_app_ (■) and secretory *P*_app_ (■) of rhodamine-123 with LC478 (1 and 10 µM) or verapamil (10 µM) across Caco-2 cells. Vertical bars represent standard deviation (SD) and the number of each group is three. * Significantly different (*p* < 0.05) from absorptive (M to S) group. ^#^ Significantly different (*p* < 0.05) from the control group. (**B**) The *EC*_50_ curves of verapamil (left) and LC478 (right) on rhodamine-123 accumulation in Caco-2 cells. Vertical bar represents standard error of the mean (SEM) and number of each group is three.

To examine the effect of LC478 on P-gp activity, rhodamine-123 transmembrane efflux was conducted in Caco-2 cells. First, transmembrane efflux of rhodamine-123 in Caco-2 cells was evaluated in the presence of LC478 (1 or 10 µM) or verapamil (10 µM), a representative P-gp inhibitor (Figure 2A and Table 1). Verapamil (10 µM), a positive control, efficiently inhibited P-gp mediated efflux of rhodamine-123 represented by secretory *P*_app_ values, and its activity was decreased to 136% of the control. LC478 also exhibited the significant inhibitory effects (45.3% decrease by 1 µM LC478 and 169% decrease by 10 µM LC478, respectively) based on secretory *P*_app_ values, indicating that LC478 concentration dependently inhibited P-gp mediated efflux activity. Efflux ratios of LC478 (10 µM) and verapamil (10 µM) were 0.349- and 0.362-fold lower than that of control, respectively. However, there was no changes of absorptive *P*_app_ values among all groups.

In addition, the half-maximal inhibitory constant (*IC*_50_) values of verapamil or LC478 for inhibition of P-gp activity were estimated corresponding to their half-maximal effective constant (*EC*_50_) values for increasing rhodamine-123 accumulation. The *EC*_50_ curves of verapamil and LC478 for percentage of rhodamine-123 accumulation in Caco-2 cells were shown in Figure 2B. The *IC*_50_ values of verapamil and LC478 for P-gp activity were 2.874 ± 0.432 and 1.674 ± 0.404 µM in Caco-2 cells, based on their respective EC_50_ value for increasing rhodamine accumulation in Caco-2 cells.

### 3.3. Effect of LC478 on P-gp Mediated Efflux of Docetaxel in Caco-2 Cells

**Figure 3:**
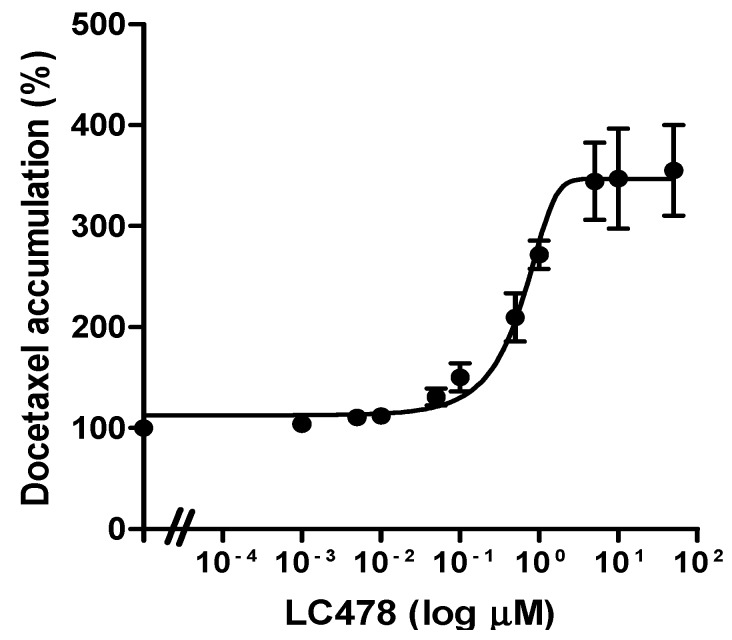
Effect of LC478 on P-gp activity. The *EC*_50_ curves of LC478 on docetaxel accumulation in Caco-2 cells. The vertical bar represents SEM and the number of each group is three.

To determine the effect of LC478 on P-gp mediated efflux of docetaxel in Caco-2 cells, the *IC*_50_ value of LC478 on P-gp mediated efflux of docetaxel was estimated from *EC*_50_ value of LC478 for increasing docetaxel accumulation in Caco-2 cells. The *EC*_50_ curves of LC478 for percentage of docetaxel accumulation in Caco-2 cells were shown in Figure 3. The *IC*_50_ of LC478 for the P-gp activity were 0.601 ± 0.115 µM in Caco-2 cells based on its *EC*_50_ value for increasing docetaxel accumulation in Caco-2 cells.

### 3.4. Effect of LC478 on Pharmacokinetics of Docetaxel

**Figure 4:**
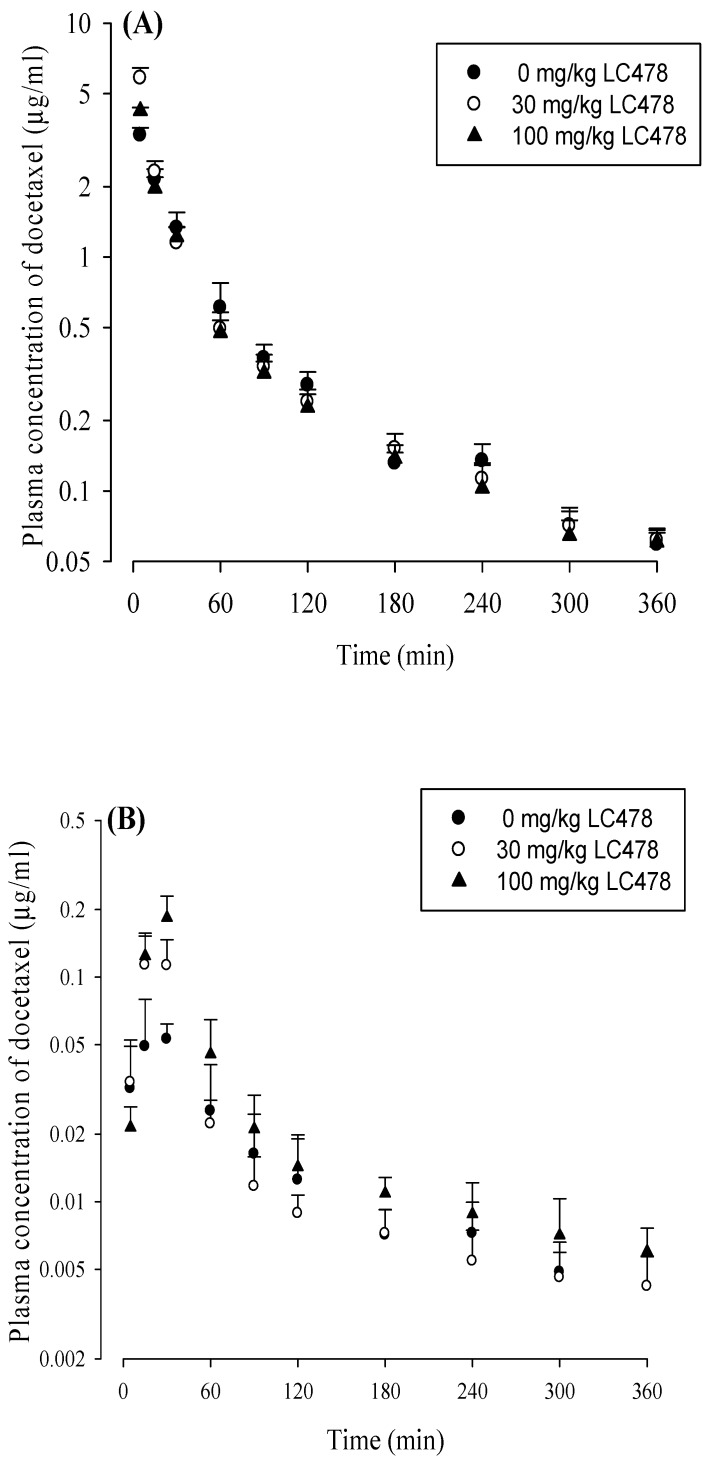
(**A**) Mean arterial plasma concentration–time profiles of docetaxel after its intravenous administration with 0 (○; *n* = 6), 30 (●; *n* = 6) or 100 (▲; *n* = 6) mg/kg LC478 in rats. (**B**) Mean arterial plasma concentration–time profiles of docetaxel after its oral administration with 0 (○; *n* = 5), 30 (●; *n* = 6), and 100 (▲; *n* = 6) mg/kg LC478 in rats. Vertical bar represents SD.

The mean arterial plasma concentration−time profiles of docetaxel after intravenous and oral administration of docetaxel with oral LC478 (0, 30 or 100 mg/kg) to rats are shown in Figure 4. The relevant pharmacokinetic parameters are also summarized in Table 2. After intravenous administration of docetaxel with 30 or 100 mg/kg LC478, pharmacokinetic parameters of docetaxel (except *CL*_R_ and *Ae*_0–24 h_) did not differ from those without LC478. The *CL*_R_ of docetaxel with 100 mg/kg LC478 was significantly slower (by 40%) than that without LC478. In addition, *Ae*_0–24 h_ of docetaxel with 100 mg/kg LC478 was significantly smaller (by 31.1% and 37.2%) than that with 0 and 30 mg/kg LC478, respectively.

After oral administration of docetaxel with 100 mg/kg LC478, pharmacokinetic parameters of docetaxel significantly changed as following: *AUC*_last_ was significantly greater (by 94.6% and 51.6%), *AUC*_inf_ was significantly greater (by 109% and 57.1%), *C*_max_ was significantly higher (by 216% and 48.4%), *CL*/*F* was significantly slower (by 40.7% and 43.1%), *V*_z_/*F* was significantly smaller (by 29.7% and 40.7%), *CL*_R_ was significantly slower (by 48.9% and 47.8%), and *GI*_24 h_ was significantly smaller (by 52% and 45.2%) than those with 0 and 30 mg/kg LC478, respectively. In the case of oral administration of docetaxel with 30 mg/kg LC478, pharmacokinetic parameters of docetaxel, except *C*_max_, were not changed compared to those without LC478. The *C*_max_ values of docetaxel with 0, 30, and 100 mg/kg LC478 were significantly different from each other. The *F* and *F*_rel_ of docetaxel with 100 mg/kg LC478 were considerably increased (by 102% and 109%) compared to those without LC478.

### 3.5. Effect of LC478 on Bi-Directional Transport of Rhodamine-123 or Docetaxel across Small Intestine in Rats

**Figure 5:**
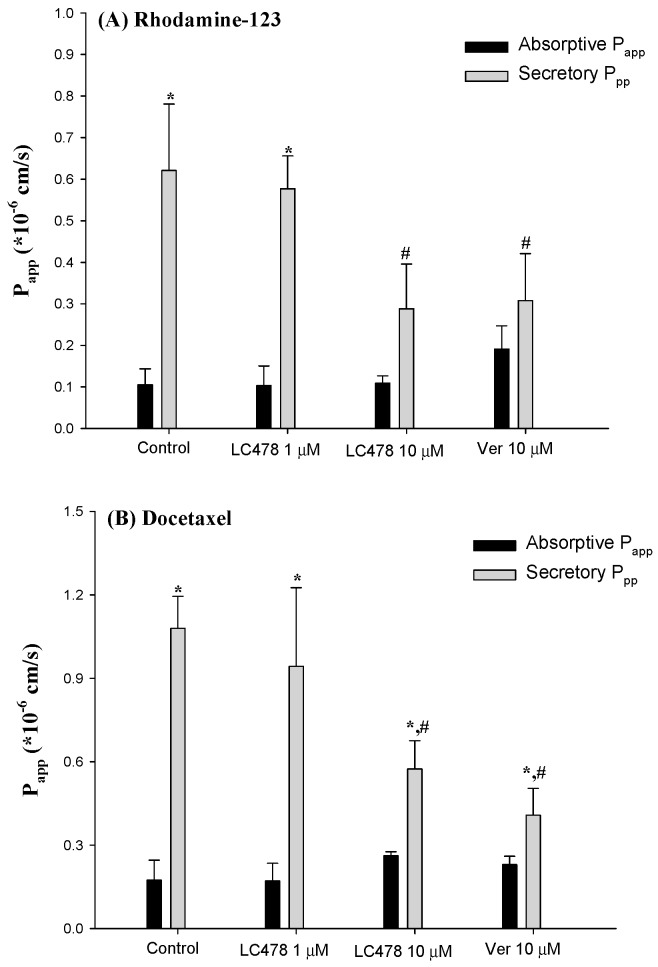
Absorptive *P*_app_ (■) and secretory *P*_app_ (■) of Rhodamine-123 (**A**) or docetaxel (**B**) across the rat small intestine with various concentrations of verapamil or LC478. Vertical bar represents SD. * Significantly different (*p* < 0.05) from absorptive (M to S) group. ^#^ Significantly different (*p* < 0.05) from control group.

Effect of LC478 on *P*_app_ values and efflux ratios from bi-directional transport of rhodamine-123 and docetaxel across small intestine are shown in Figure 5 and Table 3. First, rhodamine-123 transport across small intestine was apically polarized because secretory *P*_app_ (S to M) value was 5.99-fold higher than absorptive *P*_app_ (M to S) in the control group. In the presence of 10 µM verapamil, the secretory *P*_app_ of rhodamine-123 significantly decreased (by 104%) and consequently, the apically directed polarity in rhodamine-123 transport was considerably attenuated (efflux ratio: 6.21→1.70) compared with the control group. This suggests that the apically polarized transport of rhodamine-123 across the small intestine is predominantly facilitated by P-gp rather than other efflux transporters. Interestingly, the apically directed polarity in rhodamine-123 transport with 10 µM LC478 was considerably attenuated by secretory *P*_app_ reduction (by 118%) compared with the control group. These results suggest that P-gp mediated efflux activity of rhodamine-123 in the small intestine was significantly attenuated by LC478.

We observed a similar tendency with bi-directional transport of docetaxel. In the control group, secretory Papp was 6.21-fold higher than absorptive *P*_app_, indicating that docetaxel transport across the small intestine was apically polarized. In the presence of 10 µM verapamil or LC478, the secretory *P*_app_ of docetaxel significantly decreased (by 165 or 88.2%), indicating that apically polarized transport of docetaxel across the small intestine was considerably attenuated by LC478. Consequently, the efflux ratios of docetaxel with 10 µM verapamil or 10 µM LC478 were reduced compared with those in the control group (efflux ratio changes: 6.79→2.00 or 6.79→2.19), suggesting that P-gp mediated docetaxel efflux across the small intestine might be inhibited by LC478.

### 3.6. Effect of LC478 for Docetaxel Metabolism in Liver and Small Intestine

**Figure 6:**
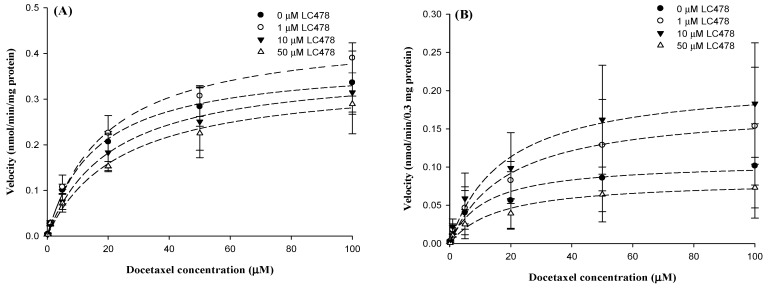
The graph for velocity versus docetaxel concentrations in hepatic (**A**) and intestinal (**B**) microsomes. Symbols represent LC478 concentrations: 0 (●; *n* = 3), 1 (○; *n* = 3), 10 (▼; *n* = 3) and 50 (∆; *n* = 3) μM LC478. Vertical bar represents SD.

We investigated the effect of LC478 on docetaxel metabolism by examining kinetics for docetaxel disappearance with various concentrations of LC478 in rat hepatic and intestinal microsomes (Figure 6 and Table 4). The *V*_max_ values of docetaxel were similar regardless of LC478 concentrations in hepatic microsomes, suggesting that the maximum velocity for the disappearance (primarily metabolism) of docetaxel was not influenced by LC478. Furthermore, the constant *K*_m_s of docetaxel with and without LC478 in the hepatic microsomes suggested that the enzyme(s) affinity with docetaxel was not altered by LC478. As a result, LC478 did not change *CL*_int_ of docetaxel in hepatic microsomes. LC478 also did not later *V*_max_, *K*_m_, and *CL*_int_s of docetaxel in intestinal microsomes. Thus, hepatic and intestinal metabolism of docetaxel were not inhibited by LC478 in this study.

### 3.7. Effect of LC478 on Rat Plasma Protein Binding of Docetaxel Using Equilibrium Dialysis

Bound fractions of docetaxel with 0, 0.1 and 10 μg/mL LC478 to fresh rat plasma were 30.6% ± 11.5%, 33.4% ± 3.59%, and 29.7% ± 10.1%, respectively. LC478 did not affect the protein binding of docetaxel in rat plasma.

## 4. Discussion

It has been reported that the absorbed fractions of oral dose in the gastrointestinal tract is well correlated between rats and humans [49]. There are similar levels of P-gp expression between rat mdr1a and human MDR1 and overlapping substrate specificity with similar affinity for numerous P-gp substrates [50]. Moreover, sequential homologies of CYPs are high (more than 65% for CYP3A subfamily) between rats and humans [51], and conserved regions for P450 reductase, heme and signal peptide generally increase this similarity [31]. Therefore, in vivo and in vitro results using rats in this study can provide a meaningful forecast for changes in oral absorption and pharmacokinetics of docetaxel by co-administration of LC478 in humans.

LC478 showed potent inhibitory effects on P-gp mediated efflux along with CYP3A-mediated metabolism of paclitaxel without unexpected intrinsic side effects from MES-SA/DX5 sarcoma cells [34]. After the negligible intrinsic cytotoxicity of LC478 (0.001–100 μM) for 24 h in Caco-2 cells was confirmed, the effect of LC478 on P-gp activity was investigated. Firstly, *IC*_50_ of LC478 for P-gp activity (represented by *EC*_50_ of LC478 for increasing rhodamine-123 accumulation) in Caco-2 cells (2.78 μM) was similar to that of verapamil (1.67 μM) in Figure 2B, indicating that LC478 inhibited P-gp activity in Caco-2 cells. In addition, the *IC*_50_ of LC478, 0.601 μM, for P-gp mediated efflux of docetaxel in Caco-2 cells (Figure 3) suggested that LC478 has the potential to overcome the intestinal absorption of docetaxel via P-gp efflux.

In in vivo pharmacokinetic studies, sufficient concentration of P-gp inhibitor is required to inhibit P-gp mediated efflux on target sites (e.g., intestine) [37,52]. However, there was no information about LC478 pharmacokinetics and then 30 and 100 mg/kg of LC478 were arbitrarily adjusted to investigate the effect of LC478 on P-gp inhibition. In case of docetaxel, 20 mg/kg of docetaxel was selected because 2–20 mg/kg and 20–100 mg/kg of docetaxel showed the linear pharmacokinetic profiles in rats [37].

The contribution of gastrointestinal (including biliary) excretion of unchanged docetaxel to *CL*_NR_ of the drug was almost negligible; the *GI*_24 h_ was less than 1.76% of the intravenous dose (Table 2). Similarly, it has been reported that docetaxel is mainly eliminated via hepatic metabolism and the biliary excretion of docetaxel as a parent form into feces was negligible [13]. Thus, the *CL*_NR_ of docetaxel (Table 2) could represent its metabolic clearance. In the intravenous study, unchanged *CL*_NR_ of docetaxel without LC478 compared to those with 30 and 100 mg/kg LC478 (Table 2) suggested that LC478 did not alter docetaxel metabolism.

Because docetaxel is a low hepatic excretion drug [53], its hepatic clearance (metabolism) depends on the hepatic intrinsic clearance and the free (unbound to plasma proteins) fraction in the plasma [54]. At this point, the unchanged *CL*_NR_ of docetaxel with 30 or 100 mg/kg LC478 (Table 2) could have been supported by in vitro unchanged hepatic *CL*_int_ of docetaxel and free fraction of docetaxel in plasma without LC478 compared to those with LC478 (Table 4). Additionally, *K*_m_ and *V*_max_ values of docetaxel with 1, 5, and 10 μM LC478 were not changed compared to those without LC478 in in vitro hepatic microsomal studies (Table 4), indicating that LC478 did not affect the affinity between metabolic enzyme and docetaxel as well as the maximum rate of the metabolism of docetaxel.

In renal excretion of docetaxel, *Ae*_0–24h_ of docetaxel was a very small portion of the docetaxel dose (less than 1.56% of dose; Table 2). Despite the negligible contribution of *CL*_R_ to *CL* (less than 1.56%; Table 2), LC478 seemed to affect renal excretion of docetaxel in rats. Interestingly, the estimated *CL*_R_ of docetaxel considering the free fractions of docetaxel in the plasma (*CL*_R_,_fu_) values are 5.04, 5.15, and 2.55 mL/min/kg with 0, 30, and 100 mg/kg of LC478, respectively. The *CL*_R,fu_ of docetaxel without LC478 was similar to the reported glomerular filtration rate (GRF, represented by creatinine clearance, 5.24 mL/min/kg) in rats [55], indicating that docetaxel is excreted via glomerular filtration into urine. Furthermore, 30 mg/kg LC478 seemed not to change the mechanism of renal excretion of docetaxel because *CL*_R_,_fu_ of docetaxel with 30 mg/kg LC478 was as similar as creatinine clearance. However, *CL*_R_,_fu_ of docetaxel with 100 mg/kg LC478, 2.55 mL/min/kg, slowed compared to the creatinine clearance in rats [10], indicating that 100 mg/kg of LC478 might incur reabsorption of docetaxel in rat’s renal tubules. Although 100 mg/kg LC478 increased renal reabsorption of docetaxel, it did not affect the systemic exposure (e.g., *AUC*) of docetaxel (Table 2).

In the oral study, the greater *AUC*_inf_, *F* and *F*_rel_ of docetaxel with 100 mg/kg LC478 than those with 0 and 30 mg/kg of LC478 (Table 2) suggested that LC478 might inhibit P-gp mediated efflux and/or metabolism of docetaxel in intestine. Although the incomplete docetaxel from the gastrointestinal tract in rats brought the extremely low *F*_rel_s of docetaxel (Table 2), the absorbed fractions of docetaxel were differently changed by co-administration with various doses of LC478. For comparison, we estimated the mean “true” unabsorbed fractions (*F*_unabs_) after oral docetaxel to rats without and with LC478 based on the following reported equation [49];

0.417 = *F*_unabs_ + (0.00881 × 0.0197)    0 mg/kg oral LC478 
0.365 = *F*_unabs_ + (0.0101 × 0.0229)    30 mg/kg oral LC478 
0.200 = *F*_unabs_ + (0.0176 × 0.0396)    100 mg/kg oral LC478 (5)

in which, 0.417 (0.365 and 0.200), 0.00881 (0.0101 and 0.0176), and 0.0197 (0.0229 and 0.0396) are the *GI*_24 h_ after oral and intravenous administration, and *F*, respectively, with 0 mg/kg LC478 (30 and 100 mg/kg LC478). The ‘*F*_unabs_’ values thus estimated were 41.7, 36.5, and 20.0% for with 0, 30, and 100 mg/kg of LC478, respectively. Thus, the increased gastrointestinal absorption of oral docetaxel with 100 mg/kg LC478 could be responsible for the greater *AUC*_inf_ of docetaxel than those with 0 and 30 mg/kg LC478 (Table 2). Especially, the ratio of *AUC* with inhibitor (*AUC*_i_)/*AUC* without inhibitor (*AUC*_0_) >1.25 is classified as the relevant drug interaction by inhibitor in U.S. FDA criteria [27,40]. In Table 2, *F*_rel_ values in rats with 100 mg/kg LC478, 209%, supported that 100 mg/kg LC478 caused pharmacokinetic interaction of docetaxel via P-gp inhibition.

To further investigate the mechanism for the enhanced gastrointestinal absorption of docetaxel by LC478, the ussing chamber studies the rat’s small intestine was performed. It has been reported that P-gp mediated efflux involves the apically-polarized transport of docetaxel [29,32]. In Table 2 and Figure 4, LC478 or verapamil did not alter the apically polarized transport of rhodamine-123, a representative P-gp substrate, or docetaxel across small intestine in rats. However, the secretary permeability of rhodamine-123 or docetaxel was considerably attenuated by 10 µM verapamil and 10 µM of LC478. These results suggest that LC478 could enhance the gastrointestinal absorption of docetaxel at least partly by inhibiting P-gp mediated efflux. Moreover, only 10 µM LC478 reduced the secretary transport of rhodamine-123 or docetaxel across the small intestine, indicating that a sufficient concentration of LC478 (10 µM) might be required to inhibit P-gp mediated efflux of docetaxel. LC478 concentrations in the intestine after oral administration of 30 and 100 mg/kg LC478 were 3.25–5.18 µM and 9.37–12.3 µM (our unpublished data), respectively. Namely that LC478 concentrations in the intestine from oral administration of 100 mg/kg LC478 were close to 10 µM, which might sufficiently inhibit the intestinal P-gp activities to efflux docetaxel. 

According to the FDA criteria [56,57], the [I]/*IC*_50_ value was used to predict the P-gp inhibition related drug interaction. In this case, [I] represents gut concentration of the inhibitor and [I]/*IC*_50_ >10 is considered as the P-gp inhibition-mediated drug interaction happens in intestinal tract [56,57]. When [I] is LC478 concentration in the intestine after oral administration of 100 mg/kg LC478, 9.37–12.3 µM (our unpublished data) and *IC*_50_ is 0.601 μM (Figure 3), [I]/*IC*_50_ values were 15.6–20.5. These estimated values suggested that a sufficient concentration of LC478 remains in intestinal tract when 100 mg/kg of LC478 was orally administered. However, [I]/*IC*_50_ values from oral administration of 30 mg/kg LC478 were 5.41–8.62. This result also supported that only 100 mg/kg of LC478 enhanced the gastrointestinal absorption of docetaxel; the absorbed fraction of docetaxel in the gastrointestinal tract with 100 mg/kg LC478 was larger than those with 0 and 30 mg/kg LC478 (0.801 versus 0.583 and 0.635, respectively). Thus, a sufficient amount (or concentration) of LC478 seemed to be required to inhibit P-gp for docetaxel absorption.

In the aspect of metabolism, there was no change of *CL*_int_ values of docetaxel with 1, 5, and 10 μM LC478 compared to that of LC478 alone in vitro intestinal microsomal study (Table 4), supporting that LC478 did not affect intestinal metabolism of docetaxel. Therefore, the greater *AUC*, *F* and *F*_rel_ after oral docetaxel administration with 100 mg/kg LC478 were because that LC478 sufficiently increased intestinal absorption of docetaxel via P-gp inhibition.

## 5. Conclusions

LC478 (100 mg/kg, not 30 mg/kg) increased intestinal absorption of docetaxel via P-gp inhibition, leading to an increase in oral bioavailability of docetaxel in rats. These results demonstrated the feasibility of LC478 as an ideal bioavailability enhancer for drugs with low absorption manners. Further toxicological and clinical evaluations of LC478 will confer a potential of LC478 as a P-gp inhibitor.

## Figures and Tables

**Table 1:** Absorptive *P*_app_, secretory *P*_app_, efflux ratio of rhodamine-123 in Caco-2 cells with various concentrations of LC478 or verapamil.

Compounds	*P*_app_ (× 10^−6^ cm/s)		Efflux Ratio
	Absorptive (A to B) *n* = 3	Secretory (B to A) *n* = 3	
Control	0.231 ± 0.0388	1.50 ± 0.113 ^a^	6.49
1 μM LC478	0.226 ± 0.0429	1.03 ± 0.264 ^a,b^	4.56
10 μM LC478	0.245 ± 0.0469	0.555 ± 0.0564 ^a,b^	2.27
10 μM Verapamil	0.270 ± 0.0634	0.634 ± 0.207 ^a,b^	2.35

Data are the mean ± SD. *P*_app_, permeability coefficient; efflux ratio, the ratio of secretory *P*_app_ to absorptive *P*_app_. ^a^ Significantly different (*p* < 0.05) from absorptive (A to B) group. ^b^ Significantly different (*p* < 0.05) from the control group.

**Table 2:** Pharmacokinetic parameters of docetaxel after intravenous and oral administration of docetaxel (20 mg/kg) with 0, 30, or 100 mg/kg of LC478 in rats.

Parameter	0 mg/kg	30 mg/kg	100 mg/kg
Intravenous Study	*n* = 6	*n* = 6	*n* = 6
Body weight (g)	302 ± 7.45	296 ± 4.49	301 ± 18.8
*AUC*_last_ (µg min/mL)	253 ± 38.1	290 ± 46.6	268 ± 39.2
*AUC*_inf_ (µg min/mL)	265 ± 39.0	303 ± 46.5	275 ± 40.2
Terminal half-life (min)	122 ± 36.6	144 ± 37.6	151 ± 34.5
*MRT* (min)	57.9 ± 4.63	58.0 ± 11.5	67.1 ± 14.6
*CL* (mL/min/kg)	78.4 ± 13.1	67.9 ± 11.7	70.4 ± 9.10
*CL*_R_ (mL/min/kg) ^a^	1.13 ± 0.431	1.06 ± 0.235	0.678 ± 0.253
*CL*_NR_ (mL/min/kg)	77.2 ± 12.8	66.8 ± 11.4	69.7 ± 9.13
*V*_ss_ (L/kg)	4.57 ± 1.03	4.02 ± 1.36	4.91 ± 1.44
*Ae*_0–24 h_ (% of dose) ^b^	1.42 ± 0.418	1.56 ± 0.129	0.979 ± 0.365
*GI*_24 h_ (% of dose)	0.881 ± 0.411	1.01 ± 0.731	1.76 ± 0.958
*F*_rel_ (%)	-	114	104
Oral Study	*n* = 5	*n* = 6	*n* = 6
Body weight (g)	247 ± 2.74	244 ± 6.07	253 ± 8.54
*AUC*_last_ (µg min/mL) ^b^	4.97 ± 1.44	6.38 ± 1.60	9.67 ± 2.05
*AUC*_inf_ (µg min/mL) ^b^	5.21 ± 1.27	6.94 ± 1.74	10.9 ± 3.02
*C*_max_ (µg/mL) ^c^	0.0583 ± 0.0207	0.124 ± 0.446	0.184 ± 0.459
*T*_max_ (min)	15 (5−60)	30 (15−30)	30 (15−30)
*CL*/*F* (mL/min/kg) ^b^	3039 ± 805	3167 ± 1021	1803 ± 515
*V*_z_/*F* (L/kg) ^b^	670 ± 170	794 ± 175	471 ± 117
*CL*_R_ (mL/min/kg) ^b^	3.50 ± 1.10	3.43 ± 1.15	1.79 ± 0.474
*Ae*_0–24 h_ (% of dose)	0.103 ± 0.0510	0.104 ± 0.0362	0.0870 ± 0.0383
*GI*_24 h_ (% of dose) ^b^	41.7 ± 13.3	36.5 ± 12.6	20.0 ± 7.44
*F*_abs_ (%)	58.3	63.5	80.1
*F* (%)	1.97	2.29	3.96
*F*_rel_ (%)	-	133	209

Data are the mean ± SD. *AUC*_last_, total area under the plasma concentration–time curve from time zero to the time of last blood sampling point; *AUC*_inf_, total area under the plasma concentration–time curve from time zero to infinity; *MRT*, mean residence time; *CL*, time-averaged total body clearance; *CL*_R_, time-averaged renal clearance; *CL*_NR_, time-averaged non-renal clearance; *V*_ss_, apparent volume of distribution at steady state; *Ae*_0–24 h_, percentage of the dose excreted in the urine up to 24 h; *GI*_24 h_, percentage of the dose recovered from the gastrointestinal tract (including its contents and feces) at 24 h; *C*_max_, peak plasma concentration of docetaxel; *T*_max_, time to reach *C*_max_; *CL*/*F*, apparent oral clearance; *V*_z_/*F*, apparent volume of distribution during elimination; *F*_abs_, absorbed fraction of oral dose; *F*, extent of absolute oral bioavailability; *F*_rel_, extent of relative bioavailability. ^a^ 100 mg/kg LC478 was significantly different (*p* < 0.05) from 0 mg/kg LC478, but not 30 mg/kg LC478. ^b^ 100 mg/kg LC478 was significantly different (*p* < 0.05) from 0 and 30 mg/kg LC478. ^c^ All group were significantly different (*p* < 0.05) from each other.

**Table 3:** Absorptive *P*_app_, secretory *P*_app_, efflux ratio of rhodamine-123 or docetaxel across the small intestine with various concentrations of LC478 or verapamil.

Compounds	*P*_app_ (× 10^−6^ cm/s)		Efflux Ratio
	Absorptive (M to S)	Secretory (S to M)	
Rhodamine-123	*n* = 3	*n* = 3	
Control	0.105 ± 0.0387	0.629 ± 0.160 ^a^	5.99
1 µM LC478	0.103 ± 0.0472	0.577 ± 0.0791 ^a^	5.60
10 µM LC478	0.188 ± 0.0177	0.288 ± 0.108 ^b^	1.53
10 µM verapamil	0.191 ± 0.0562	0.308 ± 0.113 ^b^	1.61
Docetaxel	*n* = 3	*n* = 3	
Control	0.174 ± 0.0721	1.08 ± 0.115 ^a^	6.21
1 µM LC478	0.171 ± 0.0639	0.943 ± 0.283 ^a^	5.51
10 µM LC478	0.262 ± 0.0139 ^b^	0.574 ± 0.101 ^a,b^	2.19
10 µM verapamil	0.230 ± 0.0303 ^b^	0.408 ± 0.0959 ^a,b^	1.77

Data are the mean ± SD. *P*_app_, permeability coefficient; efflux ratio, the ratio of secretory *P*_app_ to absorptive *P*_app_. ^a^ Significantly different (*p* < 0.05) from absorptive (M to S) group. ^b^ Significantly different (*p* < 0.05) from control group.

**Table 4:** *K*_m_, *V*_max_, and *CL*_int_ for the disappearance of docetaxel with or without LC478 in hepatic and intestinal microsomes.

Parameters	Concentrations of LC478 (µM)			
	0	1	5	10
Hepatic microcomes	*n* = 3	*n* = 3	*n* = 3	*n* = 3
*K*_m_ (µM)	20.2 ± 2.78	20.6 ± 2.09	21.4 ± 2.65	23.1 ± 1.74
*V*_max_ (nmol/min/mg protein)	0.464 ± 0.107	0.461 ± 0.0474	0.438 ± 0.0578	0.427 ± 0.0411
*CL*_int_ (µL/min/mg protein)	0.0229 ± 0.00204	0.0226 ± 0.00454	0.0206 ± 0.00280	0.0187 ± 0.00314
Intestinal microsomes	*n* = 3	*n* = 3	*n* = 3	*n* = 3
*K*_m_ (µM)	18.1 ± 1.20	18.3 ± 7.07	20.2 ± 8.94	17.4 ± 1.01
*V*_max_ (nmol/min/0.3 mg protein)	0.109 ± 0.0502	0.133 ± 0.0343	0.133 ± 0.0737	0.105 ± 0.0355
*CL*_int_ (µL/min/0.3 mg protein)	0.00603 ± 0.00291	0.00767 ± 0.00158	0.00757 ± 0.00559	0.00688 ± 0.00349

Data are the mean ± SD. *K*_m_, the concentration at which the rate is one-half of the *V*_max_; *V*_max_, maximum velocity; *CL*_int_, intrinsic clearance.
